# Infertility and Its Treatments in Association with Autism Spectrum Disorders: A Review and Results from the CHARGE Study

**DOI:** 10.3390/ijerph10083715

**Published:** 2013-08-19

**Authors:** Kristen Lyall, Alice Baker, Irva Hertz-Picciotto, Cheryl K. Walker

**Affiliations:** 1 Department of Public Health Sciences, University of California Davis, One Shields Ave, Med-Sci 1C, Davis, CA 95616, USA; E-Mail: ihp@phs.ucdavis.edu; 2 University of California Davis MIND Institute, 2825 50th Street, Sacramento, CA 95817, USA; E-Mails: alicebaker77@gmail.com (A.B.); ckwalker@phs.ucdavis.edu (C.K.W.); 3 Department of Obstetrics and Gynecology, University of California Davis Health System, 4860 Y St., Suite 2500, Sacramento, CA 95817, USA

**Keywords:** infertility, autism, ASD, assisted reproductive technology, infertility therapies

## Abstract

Previous findings on relationships between infertility, infertility therapies, and autism spectrum disorders (ASD) have been inconsistent. The goals of this study are first, to briefly review this evidence and second, to examine infertility and its treatments in association with having a child with ASD in newly analyzed data. In review, we identified 14 studies published as of May 2013 investigating infertility and/or its treatments and ASD. Overall, prior results showed little support for a strong association, though some increases in risk with specific treatments were found; many limitations were noted. In new analyses of the CHildhood Autism Risk from Genetics and the Environment (CHARGE) population-based study, cases with autism spectrum disorder (ASD, n = 513) and controls confirmed to have typical development (n = 388) were compared with regard to frequencies of infertility diagnoses and treatments overall and by type. Infertility diagnoses and treatments were also grouped to explore potential underlying pathways. Logistic regression was used to obtain crude and adjusted odds ratios overall and, in secondary analyses, stratified by maternal age (≥35 years) and diagnostic subgroups. No differences in infertility, infertility treatments, or hypothesized underlying pathways were found between cases and controls in crude or adjusted analyses. Numbers were small for rarer therapies and in subgroup analyses; thus the potential for modest associations in specific subsets cannot be ruled out. However, converging evidence from this and other studies suggests that assisted reproductive technology is not a strong independent risk factor for ASD. Recommendations for future studies of this topic are provided.

## 1. Introduction

Autism spectrum disorders (ASD) are developmental conditions characterized by restrictive, repetitive behaviors and deficits in communication and social interaction. Though genetic factors are known to be involved in the etiology of ASD, research demonstrates that environmental factors play a crucial role as well [1,2]. For example, congenital rubella [3], or maternal use of thalidomide [4] or valproic acid [5] during pregnancy can lead to autistic behaviors in the offspring, and many pre- or perinatal maternal factors, including obstetric complications and gestational diabetes, have been associated with ASD [6,7]. 

A number of studies have investigated maternal infertility, typically defined as failure to get pregnant after 12 months or more of unprotected intercourse without success [8], and use of infertility treatments in association with ASD. These factors are attractive targets, given the trend over recent decades toward increased parental age (which has been associated with both use of infertility treatments and having a child with ASD), and the parallel rises in use of infertility treatment and in ASD prevalence [9,10,11]. In addition, infertility and its treatments are plausible risk factors for ASD, given potential unknown biological effects. While the majority of prior work has suggested the general safety of infertility treatments [12], associations with multiple births, low birth weight, pre-term birth, and less consistently, birth defects and cerebral palsy, raised concerns and questions regarding other developmental conditions [13,14,15]. The first study to suggest an association with ASD reported a higher prevalence of infertility in mothers of children with ASD; however, the study size was small, and analyses were not adjusted for possible confounders [16]. Two larger, more recent studies reported no association between infertility and ASD [17,18]. However, few studies have examined types of infertility and a range of different therapies. The potential role of confounding by indication, though difficult to determine without extremely large numbers, has also not been adequately addressed in this field.

Although some reports have suggested an increased risk of ASD or, more broadly, developmental delays with use of infertility treatments, types of therapies assessed and definitions used vary by study [16,19,20,21,22]. Associations with ASD and/or developmental delays have been reported for intracytoplasmic sperm injection (ICSI) [20], *in vitro* fertilization (IVF) [19,22], and ovulation drugs [17,18]; one study reported a decreased risk of ASD with assisted conception [23], while a number of other studies have found no evidence of increased risk of ASD with use of assisted reproductive technologies [24,25,26,27]. However, most prior studies have been case-control studies relying on retrospective reports, without rigorous confirmation of exposures, and many have only provided results for ASD grouped with other conditions, and/or have had very small case numbers, limited information on types of exposures, and did not adjust for potential confounders [20,22,24,26,27]. Other studies [17,18,28] have identified associations only in subgroups, and have adjusted for different sets of covariates, including in some studies adjustment for potential intermediate factors on the pathway between therapies and ASD. Adjustment for intermediate factors changes the research hypothesis as well as the types of covariates that require control; thus, the interpretation and stability of the associations from such analyses deserve further scrutiny. A summary of studies published through May 2013 that have included report of these topics is provided in Table 1.

The underlying causes of potential associations between infertility treatments and ASD have not been carefully examined, but may include influences of the medications or procedures themselves, of underlying infertility etiology, or the integrity of the utero-placental unit in the resulting pregnancies. For instance, reproductive hormone imbalances are central to certain types of infertility, and are indications for treatments such as the use of ovulation induction drugs. A link between hormonal factors and ASD has been suggested through hypotheses about fetal testosterone [29] and evidence for increased risk with higher maternal BMI and earlier age at menarche [30], two endocrinologically related factors. Inflammation is also thought to cause a number of types of infertility, including tubal damage from endometriosis or infection, and there is emerging evidence of an etiologic role of the immune system in autism [31]. Infertility due to maternal reproductive tract anomalies, as well as male issues, has not been previously examined in association with ASD, but each of these could point to a common upstream genetic factor or condition. Only one large study has investigated multiple types of infertility in association with ASD while examining effects of infertility treatments as well; no associations with infertility were found in that investigation [17]. Another study examined infertility due to male or female factors only, but did not have the numbers to examine male-factor infertility alone [28].

The evidence to date is inadequate for drawing conclusions about the relationship between infertility, its treatments, and ASD. In particular, there is a need for studies the utilize rigorous methods for diagnostic and exposure confirmation, adjust appropriately for confounders, and provide information on a wide range of specific infertility diagnoses and treatments. To address these gaps, we sought to examine whether different types of infertility and treatments for infertility were associated with ASD in a population-based case-control study with clinically confirmed diagnoses. We also examined potential underlying pathways in exploratory analyses. Based on prior work, we hypothesized that associations with ASD would differ according to type of treatment, and that those related to hormonal and inflammatory factors would show increased risk in association with ASD. Because our study could not address all questions and all limitations of prior studies on this topic, such as confounding by indication and examination of effects of very rare treatments and diagnoses on ASD risk, in addition to reviewing the literature and conducting our own analyses, we also provide recommendations and novel frameworks for future studies of infertility and its treatments in association with ASD.

**Table 1:** Summary of prior studies of infertility and/or infertility therapies and Autism Spectrum Disorders ^1^.

Ref.	Study type	ASD n	Exposure	Relevant finding(s)	Comments
[16]	Case-control	**Unknown ASD n**	Report of infertility	Significant increase in report of infertility among parents of patients included, and increased prevalence of gestational exposure to progesterone/estrogen compounds in patients.	**No specific results or case numbers for ASD**; 61 patients with autism and schizophrenia. Used external control sources from previous surveys. Results not adjusted for potential confounders.
[20]	Case-control	3	ICSI, IVF	Higher prevalence of ASD in ICSI group compared to overall general population prevalence estimate of ASD from that time.	Study focused on parenting stress and child health-related quality of life, but reported 3 of 87 ICSI patients had ASD. No adjusted analyses.
[15]	Meta-analysis	Multiple studies	Assisted conception	Inconsistent results for ASD, insufficient data.	No summary measure presented for ASD. See below for further description of included studies.
[17]	Cohort	6,619	IVF with or without ICSI, OID with or without insemination	No significant association with overall assisted conception in adjusted analyses; significant association with medications containing follicle stimulating hormone. OR = 1.44 (95% CI 1.16, 1.80)	Largest sample size to date, population based. Primary findings in female offspring and for ovulation drugs. Adjustment for downstream consequences (gestational age, multiplicity) of exposure may have attenuated associations. Incomplete information on types of therapies.
[18]	Nested case-control	507	Infertility, ART, IVF, AI, OID	No significant associations with treatments or infertility overall, but AI and OID associated in subgroups.	Nested case-control within the Nurses’ Health Study II cohort. AI and OID were associated with “milder forms” of ASD (Asperger syndrome and PDD-NOS) among an advanced maternal age (≥35years) subgroup. Information on AI was collected only through open-ended question.
[28]	Nested case-control	370	Infertility, infertility medications (including OID), IUI	No association with infertility or treatments in singletons, but increased risk associated with infertility, medications, and IUI in multiple births.	Nested case-control within members of Kaiser Permanente Northern California. Small sample size and wide confidence intervals for analysis of multiples; lack of detailed data on other treatment types.
*The studies below were included in the Hvidtjørn et al. meta-analysis* [15]					
[23]	Case-control	461 autism	Assisted conception	Decreased risk of autism among those with assisted conception (adj OR = 0.37, 95%CI 0.41, 0.98)	Adjustment for potential downstream factors, including parity, birth weight, and birth defect, may have biased results. Only 10 exposed cases.
[19]	Retrospective cohort	**unknown ASD n**	IVF	OR for behavioral disorders comparing children born after IVF to controls: 1.68 (95% CI 1.11, 2.53).	**No specific results or case numbers for ASD**. ASD grouped in general ‘Behavioral disorders’ (n = 336 total). Control group consisted of mothers with OID use but no IVF. Adjusted for mother’s socioeconomic position.
[21]	Case-control	206 autism	Infertility requiring medical intervention	Infertility more frequent in autism probands but not significant by Chi-squared test.	Retrospective reporting. Adjusted results for infertility alone not reported. Analyses focused on obstetric sub-optimality scores rather than individual influence of infertility; no information on infertility therapies.
[25]	Retrospective cohort	762 PDD	IVF	No increased risk of PDD (ICD-10 diagnostic code F84) in IVF compared to non-IVF children (Rate ratio 1.2, NS).	Information from Danish registry over period of 7 years. Diagnostic priority for imprinting disorders given if >1 diagnosis in registry. Results not adjusted for potential confounders.
[27]	Retrospective cohort	19 autism 3 Asperger’s	IVF and ICSI	No difference in risk of autism or Asperger’s in IVF/ICSI exposed *vs*. unexposed.	Small number of ASD cases. Adjusted results for ASD alone not shown.
[26]	Retrospective cohort	5 ASD	IVF and ICSI	No differences in neurological disabilities between IVF/ICSI twins and unexposed.	Small number of ASD cases.
[24]	Retrospective cohort	**unknown ASD n**	IVF	OR comparing IVF exposed to unexposed for “developmental disturbance” non-significant.	**No specific results or case numbers for ASD**. Focused on hospital care utilization after IVF; 20 cases in developmental disturbance group, which appears to have included PDDs and others.
[22]	Case-control	**unknown ASD n **	IVF	Children born after IVF higher risk of “suspected developmental delay” compared to controls.	**No specific results or case numbers for ASD**. Reported results for 6 most common groups of disorders, including “suspected developmental delay”; ASD presumably combined in with “other diagnoses” for which no adjusted OR were calculated.

^1^ Table includes studies published as of May 2013. ASD = Autism spectrum disorder; PDD = Pervasive developmental disorder; IVF = *In vitro* fertilization; ART = Assisted reproductive technology; ICSI = Intra-cytoplasmic sperm injection; AI = Artificial insemination; IUI = Intrauterine insemination; OID = Ovulation-inducing drugs.

## 2. Experimental Section

### 2.1. Study Population

Participants in this study are part of CHildhood Autism Risk from Genetics and the Environment (CHARGE), an on-going, large, population-based case-control study drawn from several regions of California; the details of the study have been previously described [32]. Briefly, all participating children are: (a) 24–60 months at the time of enrollment, (b) live with at least one biological parent, (c) have a parent who speaks English or Spanish, (d) born in California, and (e) live in the specified study catchment areas. We identified children with autism through the California Department of Developmental Services (DDS) and healthy controls through state birth files. Controls were frequency-matched on age, sex, and geographic area to autism cases. We confirmed DDS diagnoses of autism with the Autism Diagnostic Observation Schedule (ADOS) and Autism Diagnostic Interview-Revised (ADI-R), conducted within a few months of recruitment. All clinicians conducting assessments have achieved research reliability on the instruments they administer. We used the following definition for ASD diagnoses: meeting criteria on either the communication or social interaction domain of the ADI-R, with onset before 36 months; either meeting or being within 2 points of meeting criteria in the other domain of the ADI-R; and meeting the ASD cut-off for social and communication totals of the ADOS. In the children recruited as general population controls, the Social Communication Questionnaire (SCQ) was used to screen for autism, with a cut-off score of 15; any children scoring above this point were administered the ADOS and ADI-R (and were not included as controls). For the current analysis, controls, designated “typically developing” (TD), were defined as children recruited from the general population meeting all of the following criteria: (a) a score of 14 or lower on the SCQ (b) a score of 70 or higher on the Mullen Scales of Early Learning (MSEL), and (c) a score of 70 or higher on the Vineland Adaptive Behavior Scales (VABS). We included only those children meeting these clinical-assessment cut-offs for ASD or TD (70 cases and 29 controls did not meet these criteria and were excluded for these analyses). Of remaining children, 30 individuals (23 cases and seven controls) missing infertility and infertility treatment information were further excluded, leaving a total of 918 individuals for these analyses. 

### 2.2. Exposure Information

Information on fertility therapies was collected through a telephone administered interview with the mother, known as the Environmental Exposures Questionnaire (EEQ), through prenatal and infertility clinic medical record abstraction, and through a supplemental telephone interview related solely to infertility issues, used when medical records were not available but infertility or treatments were reported. Thus, both medical record and self-reported information were used to define the exposures of infertility and its treatments in primary analyses; among those defined as exposed, 71% had infertility noted in medical records and 81% had infertility treatments noted.

All interviews were conducted in English or Spanish by trained personnel. For self-reported information, participants were asked: “Before you became pregnant with (CHILD), was there was there a period of 12 months or more when you had regular intercourse without using any method to prevent pregnancy and did not become pregnant?” Self-reported infertility was defined according to endorsement of this question. Participants were also asked: “Have you or your partner used any procedure or medication to help you get pregnant with (CHILD)?”, with detailed probes about different types of therapies, surgeries, and medications for both male and female treatments. Endorsement of any of these treatments was defined as any self-reported infertility treatment (a full list of treatments and medications queried is provided in the Appendix). We used the Center for Disease Control and Prevention definition for assisted reproductive technology (ART): any procedure that involves manipulation of both the egg and sperm [33]. 

For medical record information, we requested maternal prenatal records from the providers of all mothers (not just those self-reporting infertility or treatments), and reviewed all records received for presence of infertility diagnoses and treatments. The majority of the study group (73% overall and 77% among cases) had medical records available. For women who disclosed fertility problems and/or treatments either on their list of medical providers or in their interview responses, we also requested records from infertility specialists. An obstetrician/gynecologist (CW) reviewed prenatal medical records and medical records from specialists, and contacted participants to resolve discrepancies between responses in interviews and recorded exposures in (or omitted from) medical records if inconsistencies arose; however, the majority of women with both sources of exposure status had consistent information. 

Infertility treatments were categorized as use of any type (binary variable) and individually by specific treatment: ART, use of ovulation—inducing drugs (OID), artificial insemination (AI), female surgical procedures, or male only procedures. Infertility diagnosis was also examined as any diagnosis, as well as by the following specific diagnoses: endometriosis, tubal factor, uterine factor, male factor, diminished ovarian reserve, ovulatory dysfunction, poly-cystic ovarian syndrome (PCOS), and unexplained infertility. Infertility diagnoses were also grouped into the following categories for analysis: (1) uterine factor infertility, fibroids, and ovarian cysts (the “structural infertility” group); (2) PCOS, anovulation, irregular periods (the “hormonal infertility” group); (3) cervical or tubal factor infertility, endometriosis (the “inflammatory infertility” group); and (4) male factor infertility and factors related only to sperm (the “male factor infertility” group). In exploratory analyses, we created groups based on potential underlying pathways that might link ASD to either treatments or infertility diagnoses and problems noted in medical records. If medical records were not available, we made assumptions based on the type of treatment given as to the underlying indication. The pathway variable for these analyses was defined using four categories: (1) Structural group: uterine factor infertility, fibroids, ovarian cysts, and the surgical procedures used to repair them; (2) Hormonal group: PCOS, anovulation, irregular periods; and treatments of hormones or medications to influence ovulation or sex steroid hormone levels (*i.e*., OID without IVF or ART procedures noted); (3) Inflammatory group: underlying conditions thought to arise from or be associated with inflammation: cervical and tubal factor infertility, endometriosis, and typical treatments for these conditions—IVF and use of injectable “heavy duty” OIDs; and finally, (4) Male-factor group: male-factor infertility and treatments only without female issues—male surgeries and medications, sperm washing, sperm aspiration procedures, intrauterine insemination (IUI), or intracytoplasmic sperm injection (ICSI). For both the pathway variable and the grouped infertility diagnosis variable, for any individuals with multiple treatments or diagnoses, we classified according to the most invasive issue to obtain mutually exclusive categories.

### 2.3. Statistical Analyses

We compared basic frequencies of infertility therapies and diagnoses between cases and controls, and conducted Chi-squared tests to compare the odds of ASD across exposure groups. Multivariable regression models were used to determine the association between the following factors and ASD: (1) infertility, (2) infertility therapies; and (3) hypothesized pathways. Analyses of infertility compared those with any infertility to those without. Due to the low prevalence of individual infertility diagnoses, we used the grouped infertility variable to examine different types of infertility; in these analyses, indicator variables were created for each of the 4 infertility groups described above, as well as one for unexplained or other infertility for individuals not fitting within any other grouping; the referent group was again those with no infertility. Parallel analyses were conducted for infertility therapies, comparing those with any infertility to those without. Analyses of individual infertility therapies with sufficient numbers compared those with that particular treatment to those without it (but who may have received others). The pathway analysis used those with no treatments or infertility as the referent group, with indicator variables for the other categories. 

For all regression models, we report the associations in odds ratios for ASD *vs.* TD, and their 95% confidence intervals. We used logistic regression, and examined adjustment for the following potential confounders (in all analyses), based on a priori knowledge: maternal and paternal age (continuous variables), maternal race/ethnicity (white, Hispanic, and other), maternal education (4 categories), insurance payment for delivery (public *vs.* private), birth order (ordinal variable), pre-pregnancy smoking status (yes/no for regular smoking prior to pregnancy), and pre-pregnancy BMI (5 categories). Assessments of individual fertility therapies also considered further adjustment for use of other therapies (binary indicator). In selection of covariates, we examined variance inflation factors and correlation of covariates to avoid multicollinearity. All models also adjusted for the study matching factors (child’s age, sex, and geographic region). 

In further analyses, we adjusted for potential biases arising from differential participation rates using weighted conditional logistic regression in Proc Survey Logistic. The weights were proportional to the inverse-probability of participation (based on recruitment group, regional area at recruitment, maternal education level, age, country of birth, insurance status at child’s delivery, and child race/ethnicity) to enhance generalizability to the source population. 

In secondary analyses, we examined associations among mothers aged 35 or older at delivery, and by diagnostic subgroup (autistic disorder and broader ASD). In sensitivity analyses, as our exposures were defined according to both self report and medical record sources, we compared results of exposures classified according to each of these sources of information in isolation. 

## 3. Results

These analyses included 537 ASD cases and 381 TD controls. Parents of children with an ASD were slightly older than TD control parents, and case mothers were slightly more likely to have had a history of smoking; other demographic and lifestyle factors were similar between the groups (Table 2). 

**Table 2:** Basic characteristics of the study population (n = 918).

	ASD cases n = 537 n (%)	TD Controls n = 381 n (%)
Maternal age			
<25	79 (15%)	66 (17%)
25–29	144 (27%)	85 (22%)
30–34	168 (31%)	139 (36%)
35+	146 (27%)	91 (24%)
Paternal age		
<25	44 (8%)	47 (12%)
25–29	105 (20%)	59 (16%)
30–34	161 (30%)	129 (34%)
35+	219 (41%)	144 (38%)
Missing	8 (1.5%)	2 (0.5%)
Birth order		
Firstborn	249 (46%)	159 (42%)
Multiple birth	27 (5%)	16 (4%)
Insurance information ^1^		
Government Program	102 (19%)	56 (15%)
Insurance	435 (81%)	322 (85%)
Missing	0	3 (0.5%)
Male child ^2^	461 (86%)	318 (83%)
Race		
Caucasian/White	319 (59%)	243 (64%)
African American	18 (3%)	11 (3%)
Asian	41 (8%)	26 (7%)
Hispanic	133 (25%)	79 (21%)
Other	26 (5%)	22 (6%)
Education		
High school or less	76 (14%)	57 (15%)
Some college	218 (41%)	125 (33%)
College degree	158 (29%)	139 (36%)
Graduate degree	82 (15%)	59 (15%)
Missing	1 (0.2%)	0 (0.3%)
Autoimmune disorders ^3^	33 (6%)	30 (8%)
Gestational diabetes	58 (11%)	29 (8%)
BMI (pre-pregnancy)		
<20	67 (12%)	45 (12%)
20–24	269 (50%)	204 (54%)
25–29	123 (23%)	91 (24%)
30+	78 (15%)	41 (11%)
History of smoking ^4^	120 (23%)	64 (18%)
History of infertility ^5^	55 (10%)	39 (10%)
Infertility treatment for index birth ^6^	49 (9%)	33 (9%)
History of infertility treatment (any) ^7^	53 (10%)	38 (10%)

^1^ Method of payment at time of delivery of child. ^2^ Male children were over-selected to match the sex ratio for autism. ^3^ Self-report of any autoimmune disorder. ^4^ Regular smoking at any point prior to child’s birth. ^5^ Overall, 75% of the total study group had medical records available; of those self-reporting infertility, 86% had medical records available; of these, 65% had infertility noted in the available record. ^6^ Of those self-reporting infertility treatments, 84% had medical records available; of these, 77% had treatments confirmed in records. As noted in the text, because we defined exposures according to both medical records and self-reported information, the overall percent noted in medical records among those defined as having infertility or treatments was 71% and 81% respectively. ^7^ Includes use in any previous cycle (16 individuals, including 5 ASD cases, 8 TD, and 3 DD; information on previous use was not specifically asked in self-report questionnaire and was usually not available from medical records, but was noted when reported or recorded).

Nine percent of both ASD cases and TD controls had used at least one type of infertility treatment for the index birth. Numbers were small for rarer types of therapies, but overall, frequencies were remarkably similar between the ASD and TD groups (Table 3). The ASD case group also did not differ from TD controls by infertility diagnosis or according to hypothesized pathways. 

**Table 3:** Infertility and infertility treatments by case status.

	ASD cases n = 537 n (%)	TD Controls n = 381 n (%)
**Infertility diagnoses**			
Any infertility	55 (10%)	39 (10%)
Diminished Ovarian Reserve	1 (0.2%)	0
Endometriosis	3 (0.6%)	1 (3%)
Ovulatory dysfunction	7 (1%)	6 (2%)
Tubal factor	8 (1.5%)	3 (1%)
Uterine factor	1 (0.2%)	2 (0.5%)
Male factor	6 (1%)	3 (0.8%)
Unexplained	11 (2%)	8 (2%)
Other	16 (3%)	14 (4%)
No diagnosis information	17 (3%)	13 (3%)
Multiple diagnoses	12 (2%)	8 (1%)
**Infertility treatments**		
Any treatment	49 (9%)	33 (9%)
ART	14 (3%)	12 (3%)
IVF	13 (2%)	12 (3%)
ICSI	4 (0.7%)	6 (1.5%)
GIFT, ZIFT, or TET	1 (0.2%)	0
Donor egg, sperm, or embryo	6 (1%)	2 (0.5%)
Frozen egg, sperm, or embryo	6 (1%)	0
Artificial Insemination	12 (2%)	11 (3%)
Male Procedures	9 (3%)	4 (2%)
Surgeries ^1^	18 (3%)	8 (2%)
Any OID	28 (5%)	25 (7%)
OID-clomiphene citrate	17 (3%)	19 (5%)
OID-injections	14 (3%)	13 (4%)
FSH	8 (2%)	10 (3%)
hCG	3 (0.6%)	2 (0.6%)
Progesterone	26 (5%)	21 (5%)
GnRH agonsist	14 (3%)	11 (3%)
Post-fertilization medications ^2^	23 (4%)	23 (6%)

FSH = follicle-stimulating hormone; hCG = human chorionic gonadotropin; GnRH = gonadotropin-releasing hormone; other acronyms defined in Table 1, the text, and/or Appendix List of Infertility Procedures and Treatments Queried.^1^ Female surgeries for problems affecting ability to get pregnant: removal of fibroids, cysts, or endometriosis. ^2^ Includes progesterone, heparin, and aspirin to improve implantation.

In adjusted analyses comparing ASD cases to TD controls, fertility therapies and infertility continued to show no association with odds of ASD (Table 4). Overall, odds ratios were all close to 1 (OR for overall infertility treatment use = 1.16, 95% CI 0.70, 1.93), though for certain therapies, confidence intervals were imprecise due to small numbers. In particular, any male treatment had only nine exposed cases in the primary analysis, and while similar point estimates were similarly elevated across subgroup analyses for this treatment type (OR approaching 2), these results were not significant. Contrary to our hypotheses, no differences were noted according to hypothesized underlying pathways (Appendix, Table S1). Results were similar across models tested, including in weighted (Model 3, Table 4) and unweighted (Model 2, Table 4) analyses, and did not materially change when using reduced models including only maternal age, child year of birth, and matching factors, or conversely, when considering further adjustment for pre-pregnancy smoking and BMI, which have been associated with both the exposures and outcome under study in some investigations [30,34,35,36]. 

**Table 4:** Odds of ASD according to infertility and treatments.

	Exposed case n	Model 1 ^1^	Model 2 ^2^	Model 3 ^3^
*Infertility*				
Any Infertility	55	0.97 (0.62, 1.53)	1.00 (0.62, 1.60)	1.04 (0.64, 1.69)
*Grouped Infertility Diagnoses*				
No infertility	475	1.0	1.0	1.0
Hormone issue	17	1.35 (0.57, 3.19)	1.40 (0.58, 3.36)	1.44 (0.55, 3.80)
Inflammation issue	12	0.65 (0.26, 1.62)	0.65 (0.26, 1.64)	0.67 (0.29, 1.54)
Male issue	6	1.28 (0.34, 4.75)	1.30 (0.34, 4.96)	2.01 (0.65, 6.20)
Other or unknown issue	19	1.09 (0.52, 2.27)	1.12 (0.53, 2.35)	1.05 (0.46, 2.37)
*Treatments*				
Any infertility treatment	49	1.10 (0.68, 1.80)	1.16 (0.70, 1.92)	1.10 (0.66, 1.83)
Surgical interventions (female)	18	1.33 (0.55, 3.19)	1.39 (0.57, 3.38)	1.07 (0.49, 2.36)
ART	14	0.97 (0.43, 2.19)	1.06 (0.46, 2.44)	1.20 (0.46, 3.13)
IVF	13	0.92 (0.40, 2.10)	0.99 (0.42, 2.31)	1.14 (0.43, 3.01)
OID (any)	28	0.92 (0.51, 1.64)	0.94 (0.52, 1.70)	0.96 (0.52, 1.77)
OID-pills	17	0.71 (0.35, 1.43)	0.72 (0.35, 1.45)	0.68 (0.32, 1.42)
OID-injections	14	0.86 (0.39, 1.92)	0.89 (0.39, 2.02)	1.02 (0.44, 2.39)
Artificial Insemination	12	0.89 (0.38, 2.12)	0.89 (0.37, 2.13)	0.83 (0.34, 2.03)
Male procedures	9	1.88 (0.55, 6.47)	1.98 (0.57, 6.92)	1.99 (0.34, 11.5)

Results for other exposures/categories with fewer than 5 individuals were non-significant. ^1^ Includes adjustment for matching factors: regional area, child sex and child age. ^2^ Adjusted for: matching factors, maternal and paternal age, maternal race and education, and insurance status at delivery. Estimates were very similar when only adjusting for maternal age and the matching factors, or with additional adjustment for maternal pre-pregnancy BMI, smoking, or birth order. Additional adjustment for use of other therapies in models assessing individual therapies further did not materially change results. ^3^ Adjusted as for Model 2, with addition of inverse-probability of sampling weights. Removal of demographic covariates from weighted models did not alter results.

In subgroup analyses among mothers of advanced age (n = 237) and by diagnostic subgroup (367 autistic disorder and 170 broader ASD), results were very similar, and again non-significant for any associations with infertility and infertility treatments (data not shown; OR for any infertility treatment in the advanced maternal age group: 1.20, 95% CI 0.56, 2.59; in the autistic disorder case group, the corresponding OR was 1.27, 95% CI 0.73, 2.20). However, it should be noted that numbers were small within these groups; only 27 cases used any fertility therapies among the advanced maternal age subgroup, with numbers for individual types of therapies around 10 or fewer. Likewise, sensitivity analyses utilizing only self-reported information, or only information from medical records, also did not demonstrate any significant associations.

## 4. Discussion

The results of this case-control study do not provide evidence for an association between fertility therapies and autism spectrum disorders. We examined a number of different types of therapies and conditions underlying the infertility being treated, and overall found remarkable similarity between ASD cases and typically developing controls. However, due to the low power to detect subtler effects in our study, we cannot exclude the potential for *modest* associations with rarer therapies or conditions. These topics should therefore be further explored in very large studies with standardized outcome ascertainment and rigorous exposure information.

A major strength of this study, and an improvement over a number of prior studies examining infertility and/or its treatments in association with ASD, is the confirmation of both case status and exposures through rigorous, gold standard measures. All children included in these analyses were evaluated at the UC Davis MIND Institute for diagnostic confirmation, and detailed interviews were conducted and medical records abstracted (in the majority of the study group) for exposure information. In contrast, none of the prior studies examining these factors have confirmed case and comparison group status at this level of detail. We also had information on a full range of infertility diagnoses and treatments, which is lacking in other studies. Our estimates of frequency of use of a wide range of therapies according to ASD status thus fill a needed gap in the literature. Despite using retrospective reporting, as had been previously utilized in a number of prior investigations, we also collected medical records in a large majority of the group for confirmation. We also carried out a thorough confounder identification strategy, whereas many of the prior studies of infertility treatments and ASD failed to adjust for even basic sociodemographic risk factors [16,20,21,22,25,27]. We further took advantage of the linkage of all our cases and controls to the population birth files that included all non-participants in order to account for potential differential participation (selection bias) through weighted analyses, which has not been done in previous case-control studies of this topic.

However, a number of limitations in our work should be noted. Despite a sample size of nearly 1,000 mother-child pairs, our study was limited by the relatively rare exposures, leading to small numbers in many categories. Thus, while we had sufficient power to detect odds ratios of at least 1.75 for treatments and diagnoses with prevalence over 5%, power was reduced to detect associations for specific therapy types with infrequent use. To date, only Hvitjorn and colleagues [17] have had adequate numbers to examine rarer therapies, but unfortunately, they did not have information on many different types of treatments. We cannot rule out bias due to participation, a common problem in case-control studies, by demographic factors that could be related to the exposures studied here; however, our use of sampling weights strove to mitigate any such biases. While we did rely on retrospective reporting, between 70–80% of our exposures were confirmed in medical records. Another potential limitation, not restricted to our own study, is the definition of infertility itself; how different couples perceive “regular intercourse” is open to interpretation, and timing, diet, lifestyle and cultural factors all may influence reported infertility in ways not related to hypothesized biological pathways.

Consistent with our results, the majority of prior work suggests that use of assisted technologies does not increase risk of adverse child outcomes (with the notable exceptions of multiple births, pre-term birth and low-birth weight). A handful of studies have suggested increased risks of autism, or developmental delay, cerebral palsy, and imprinting disorders with use of ART [15,19,22]. However, our study and four other investigations [17,18,25,27], including the largest study of ASD and assisted conception to date, with over 3,600 cases and approximately 33,000 children exposed to assisted conception [17], found no association between ART and risk of ASD specifically. We also did not see associations with IVF or other ART subtypes, though numbers were small. Two prior studies have also found no association between ASD and IVF or ICSI [25,26], while results from few others have been inconsistent for more broadly defined developmental outcomes and ART subtypes [19,22,24,27].

For other types of infertility treatments, there is limited information on associations with ASD specifically. A handful of prior studies have suggested associations with ovulation drugs or medications (three studies, each of which found associations only in different subgroup analyses) [17,18,28], specific hormones (two studies) [17,28], and artificial insemination/intrauterine insemination (two studies) [18,28]. Specifically, an investigation in the Nurses’ Health Study II found a significant association between ASD and OID in an advanced maternal age subgroup [18], which was a larger subgroup than the current study; thus, smaller numbers here could account for the differences seen. The Danish study conducted by Hvitjorn and colleagues [17] found significant associations with ASD for female offspring exposed to OID as well as for use of follicle-stimulating hormone (FSH)-containing medications, while another study saw an association with urofillitropin, a purified form of FSH, only among multiple births [28]. Given that FSH-containing medications are indicated for a range of underlying problems, the meaning of these findings is not immediately evident. We did not see an association with FSH specifically, and a larger investigation than ours will be needed to replicate results. Another recent study found no association with a general category of infertility medications (that included OID) and ASD among singleton births [28], but did find a significant association among multiple births. Our results did not differ in multiple or singleton births, though as in the work by Grether and colleagues, exposed numbers among multiples were small, thereby limiting conclusions. 

The Nurses’ investigation also saw an association with AI, which the CHARGE study did not replicate; however, the source of infertility treatment information in the Nurses’ study was not as rigorous as in the current study. We did find increased odds ratios for male treatments in our study; though non-significant, use of AI is sometimes indicated for male factors. Again, Grether and colleagues’ study found an association with IUI and ASD only among multiple births but not in singletons, providing mixed results. Given that Hvitjorn and colleagues’ definition of OID included use with and without AI, future studies should also investigate AI in association with ASD, both in singleton and multiple births.

Our analyses of these infertility treatments considered adjustment for a number of potential confounders. Prior studies examining potential effects of infertility treatments have adjusted for or stratified on multiple births in attempt to assess the effect of the treatments on the various outcomes studied, not mediated by multiplicity. For comparison to this work, we examined exposures stratified by singleton and multiple births and found that results did not differ (data not shown; nor did results materially change when adjusting for birth order, which has similar issues when considering effects of infertility and its treatments). However, conditioning on a downstream consequence of exposure can introduce bias. Another example is adjustment for birth weight, a common flaw in studies of prenatal exposures; again, we did not include birth weight in our multivariable models for this reason. Future large studies may benefit from the use of more sophisticated statistical methods, such as marginal structural models (MSMs) [37,38], to determine controlled direct effects not mediated by these factors. Alternatively, using mediator analyses [39,40] may also be useful in determining the impact of factors that may be downstream of infertility therapies, assuming confounding of the intermediate-outcome association is adequately accounted for. Given the null findings for exposures in our study, we did not see significant associations with potential mediators when we conducted such analyses (results provided in Appendix, Table S2); however, pregnancy complications, low birth weight, and multiple births had fairly large estimates of percent mediation. Little prior work has investigated underlying infertility, rather than just its treatments, in association with ASD. While two small studies reported increased prevalence of infertility among mothers of affected children [16,21], and a third study reported an association only for multiple births [28], two larger studies (one registry-based and one nested case-control) have not found associations between maternal infertility and risk of ASD [17,18]; our work is consistent with these recent findings. 

Infertility treatments have been hypothesized to influence ASD through a number of mechanisms, including hormonal influences of the medications, effects of invasive procedures on DNA methylation or other direct effects of the procedure/treatment, impaired egg quality, influences of the underlying infertility, or simply through associations with downstream consequences of the treatment (such as multiple birth, pregnancy complications, low birth weight, or pre-term birth) [15,18,41]. While we had hypothesized that hormonal or inflammatory pathways may be involved, we did not see associations with these pathways as related to infertility and its treatments. However, power was limited to detect modest associations (*i.e*., those on the order of OR = 1.5 or less), given the number of exposed cases in each of the pathway groups. Continued investigation of such pathways and groupings as conducted here may be useful in learning more about potential underlying mechanisms.

*Recommendations for future studies:* It is evident that very large studies will be needed to fully elucidate the potential effects of individual infertility treatments and underlying diagnoses on risk of ASD. Besides simply increasing numbers, future studies may also benefit from the following suggestions. Rigorous diagnostic and exposure confirmation methods and definitions are required to avoid misclassification and bias. In addition to studying infertility diagnoses and treatments, investigating related factors, such as sub-fertility and time to conception may help to shed light on whether infertility itself is associated with ASD. Models testing the effect of infertility treatments among those with infertility will also be useful in determining independent effects of treatments *versus* the indication for treatment. Further, making use of hypothesized underlying pathways, such as those proposed here, may help to determine whether groups of diagnoses and treatments point to a likely etiologic role. Finally, analyses can be improved by avoiding adjustment for downstream effects of exposures and rather considering mediators, as described above, through use of one or another type of mediation analysis (MSMs, regression models, *etc*.), all of which require thorough adjustment for confounders of the intermediate-by-outcome association. 

## 5. Conclusions

Our work and that of others highlights the need for very large studies in order to fully address the topic of infertility and its treatments in association with ASD. Overall, the evidence to date suggests that women using infertility therapies do not need to be concerned about *strong* increases in risk of ASD. However, the known risks associated with infertility therapies, such as prematurity, low birth weight infants and multiple births, remain as concerns associated with use of these therapies, and evidence suggests the need for continued long-term follow-up of children conceived using these procedures [14]. Women using these therapies appear to also be at higher risk for pregnancy complications, although this increased risk could be a result of the primary infertility and its root causes. Thus, further investigations are needed to disentangle the complex role of underlying infertility, its treatments, and possible mediators of hypothesized effects on risk of ASD. The limited power to detect modest associations in our study suggests further work may be required to (a) detect subtler risks associated with specific infertility therapies and underlying infertility pathways, and (b) better understand associations in groups such as multiple births and women with advanced maternal age, for whom these treatments and issues are more common.

## Appendix

**Table S1:** Infertility pathways in association with ASD.

	Exposed case n	Model 1	Model 2	Model 3
No fertility therapies	481	1.0	1.0	1.0
Hormonal	21	1.33 (0.64, 2.80)	1.72 (0.78, 3.78)	1.33 (0.55, 3.20)
Inflammatory	16	0.93 (0.44, 1.99)	0.92 (0.41, 2.08)	0.84 (0.40, 1.74)
Male	8	1.40 (0.42, 4.68)	1.83 (0.52, 6.42)	2.94 (0.94, 9.23)

Adjusted as in Table 4; Model 1 adjusted for study matching factors, Model 2 adjusted for study matching factors as well as maternal and paternal age, maternal race and education, and insurance status at delivery; Model 3 adjusted for Model 2 covariates as well as inverse-probability weights.

**Table S2:** Percent mediation estimates for suspected mediators of the infertility therapy-ASD association.

Potential Mediator	Estimated % Mediation ^1^	p-value ^2^
Gestational diabetes	15%	0.61
Gestational hypertension or preeclampsia	30%	0.57
Low birth weight	25%	0.72
Pre-term birth	2.6%	0.89
Multiple birth	45%	0.74

^1^ Mediation proportions estimated using the SAS Proc Mediate Macro. % Mediation is of the infertility therapy-ASD association, adjusted for matching factors, maternal and paternal age, demographic factors. Pregnancy complication models (gestational diabetes or hypertension/preeclampsia) also included adjustment for pre-pregnancy smoking and BMI, whiel perinatal models (low birth weight, pre-term birth, and multiple birth) also included adjustment for pregnancy complications. ^2^ Wald-p comparing model with the mediator to model without.

### List of Infertility Procedures and Treatments Queried

#### Procedures

Assisted Hatching (AH)
Blastocyst Transfer (from a fresh IVF cycle or a frozen cycle)
Cervical Insemination (CI or ICI)
Co-culture
Egg Recipient (of donor eggs)
Frozen Embryo Transfer (FET/CET)
Gamete IntraFallopian Transfer (GIFT)
Gestational Surrogate
*In Vitro* Fertilization, embryo transfer (IVF)
IntraUterine Insemination (IUI)
IntroCytoplamic Sperm Injection (ICSI)
MicroEpididymal/Percutaneous Epididymal Sperm Aspiration (MESA or PESA)
Pre-Implantation Genetic Diagnosis (PGD)
Pronuclear Transfer (PROST)
Sub-Zonal Insemination, Partial Zona Dissection (SUZI/SZI or PZD)
Testicular Sperm Aspiration, Extraction (TESA or TESE)
Tubal Embryo Transfer (TET)
Zygote IntraFallopian Transfer (ZIFT)

#### Treatments/Medications (Trade Name in Capitals, Followed by Generic Name)

A.P.L. Chorionic gonadotropin
AMEN Medroxyprogesterone
ANTAGON Ganirelix
AYGESTIN Norethindrone acetate
BRAVELLE Chorionic gonadotropin
CETROTIDE Cetrorelix
CLOMID Clomiphene
CRINONE Progesterone
CURRETAB Medroxyprogesterone
CYCRIN Medroxyprogesterone
ELIGARD Leuprolide
FACTREL Gonadorelin
FERTINEX Follicle-stimulating hormone
FOLLISTIM Follicle-stimulating hormone
GONAL-F Follicle-stimulating hormone
LUPRON DEPOT Leuprolide
LUPRON INJECTION Leuprolide
LUTREPULSE Gonadorelin
LUVERIS Lutropin alfa
MENOPUR Follicle-stimulating hormone
METRODIN Follicle-stimulating hormone
NOVAREL Chorionic gonadotropin
OVIDREL Chorionic gonadotropin
PARLODEL Bromocriptine
PERGONAL Follicle-stimulating hormone
PREGNYL Chorionic gonadotropin
PROFASI Chorionic gonadotropin
PROMETRIUM Progesterone
PROVERA Medroxyprogesterone
REPRONEX Follicle-stimulating hormone + Luteinizing hormone
SEROPHENE Clomiphene
SYNAREL Nafarelin
VIADUR Leuprolide
ZOLADEX Goserelin
